# The Case of Severe Wound Myiasis Caused by a Minor Injury Sustained by a Spectator During a Tip‐Cat‐Sport

**DOI:** 10.1002/ccr3.71920

**Published:** 2026-01-22

**Authors:** Jahan Esha Ishrat, Kairi Hayashi, Chowdhury Ruman Uddin, Chowdhury Nafees Uddin, Hiroshi Churei, Kenji Fueki

**Affiliations:** ^1^ Department of Masticatory Function and Health Science Graduate School of Medical and Dental Sciences, Institute of Science Tokyo Tokyo Japan; ^2^ Department of Orthodontics and Sports Dentistry Tokyo Dental Chamber Dhaka Bangladesh; ^3^ Department of Maxillofacial Surgery and Prosthetics Tokyo Dental Chamber Dhaka Bangladesh

**Keywords:** *Chrysomya bezzima*, skin graft, Tip‐Cat‐Sports, wound myiasis

## Abstract

Wound myiasis commonly occurs in tropical regions and is caused by flies laying eggs on open wounds. It is essential to raise awareness about preventive measures and treatment methods for this disease, especially because many people now visit endemic areas from non‐endemic regions. This report presents a wound myiasis case caused by *Chrysomya bezziana,* resulting from a small wound sustained by a spectator of “Tip‐Cat‐sport.” A 64‐year‐old woman was admitted to a hospital in Bangladesh with a severe facial wound infection and symptoms of fever, pain, and swelling. She sustained an injury from the tip‐cat tool while watching the sport, and the wound remained untreated. Magnetic resonance imaging was used to diagnose the infection as wound myiasis caused by *Chrysomya bezziana*. Turpentine oil was applied to the open wound to create an oxygen‐deficient environment that forces maggots (larval stage of the fly) on its surface to be removed. After removal, wound debridement was performed, followed by surgical excision of the affected tissue and skin grafting. After 2 months, the wound completely healed. We hope this case report will enhance the knowledge about wound myiasis, including preventive measures and treatment options, among those with limited awareness of this condition.

## Key Clinical Message

- This report presents a wound myiasis case caused by *Chrysomya bezziana,* resulting from a small wound sustained by a spectator of “Tip‐Cat‐sport.” The wound, infected by *Chrysomya bezziana larvae*, was treated with turpentine oil, debridement, and skin grafting. This case highlights the need for awareness of prevention and treatment in endemic areas.

## Graphical abstract

This report presents a case of wound myiasis caused by *Chrysomya bezziana* which resulting from a small wound sustained by a spectator of “Tip‐Cat‐sport.” After all maggots were removed, wound debridement followed by surgical excision of the affected tissue and a skin graft was performed. After two months, the wound completely healed.

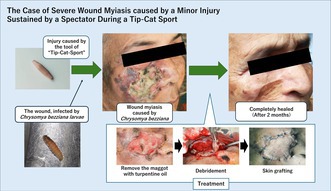

## 1 Introduction

Maxillofacial injuries have various causes, including traffic accidents and sports‐related incidents, and range in severity from minor abrasions to severe fractures [1]. Maxillofacial injuries in sports are often associated with collisions involving other players or equipment, such as balls or bats [2, 3]. However, such injuries are rarely observed among non‐players, such as spectators. Certain sports, notably baseball and cricket, pose a high risk of injury to spectators because of the nature of gameplay [4].

Maxillofacial injuries can become serious if untreated infections develop at the wound site, even if it is a minor injury. Therefore, it is essential to maintain the cleanliness of the wound and ensure that patients are kept in a sanitary environment. Some infectious diseases are specific to certain regions, and individuals traveling from other areas may have limited awareness or precautionary measures against these infections. Additionally, treatment methods may be unfamiliar in their home countries. Therefore, disseminating knowledge and treatment protocols for region‐specific diseases is of the utmost importance globally.

**FIGURE 1:**
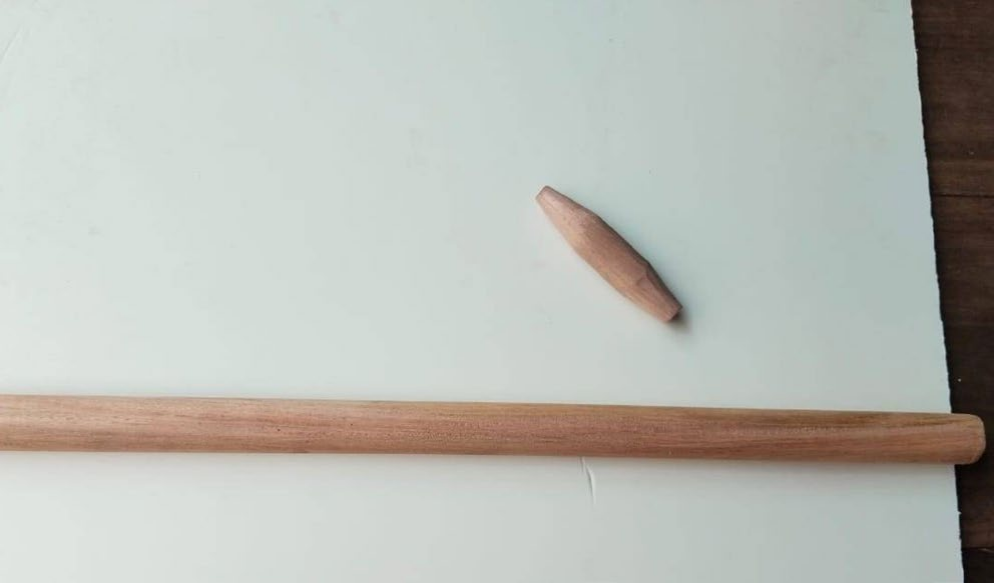
Tools of Tip‐Cat‐Sport. In Tip‐Cat‐Sport, players use a short, sharpened wooden billet and a larger stick.

In this report, we present a wound myiasis case caused by *Chrysomya bezziana*, resulting from a small wound during “Tip‐Cat‐Sport”, sustained by a spectator. This traditional sport, popular in South Asian countries, mostly Bangladesh and India, shares similarities with bat‐and‐ball games, such as baseball and cricket. In Tip‐Cat‐Sport, players use a short, sharpened wooden billet, usually no more than 8–15 cm, and a larger stick (Figure 1). The wooden billet is placed within a small circle on the ground, flicked into the air, and struck by a larger stick to move it as far as possible. The opposing player measures the distance covered by the billet through jumps and assigns points accordingly.

The term myiasis is derived from the Greek words “muia” and “iasis,” meaning “fly” and “disease” [5], and a term derived from the Greek word “myia,” meaning invasion of fly larvae into vital tissue of humans or other mammals. They can infest vertebrates, including humans, and feed on living or dead tissue as well as body fluids [6]. The term was coined by F.W. Hope in 1840 [7].

Human and animal tissues act as intermediate hosts for the *Chrysomya bezziana* larvae. In humans, the commonly affected sites are the skin, nose, ears, eyes, anus, vagina, and oral cavity [8]. Myiasis is seen globally, with a higher incidence observed in tropical and subtropical regions owing to the favorable climatic conditions of heat and humidity [8, 9].

*Chrysomya bezziana*, known as the old‐world screwworm fly, causes myiasis across many countries in Asia and Africa [10, 11, 12]. Female flies are attracted to and lay their eggs near existing wounds, where maggots hatch and feed on living tissue, causing severe injury to vertebrate hosts [13].

It is distributed throughout Africa, the Indian subcontinent, and Southeast Asia, from the People's Republic of China through the Malay Peninsula and the Indonesian and Philippine islands to Papua New Guinea [14].

Human cases of *Chrysomya bezziana* myiasis are most prevalent in India and Southeast Asia [15]. Myiasis frequently occurs in rural areas, infecting livestock. It prevails in unhealthy individuals in third‐world countries [16]. With an increasing number of people traveling to areas where wound myiasis is endemic for business, tourism, or sports tournaments, individuals are at risk of acquiring injuries that could lead to wound myiasis.

This case report aims to increase global awareness and treatment of this endemic disease contributing to the prevention of wound myiasis in a broad population of spectators and sports players.

## 2 Case History/Examination

**FIGURE 2:**
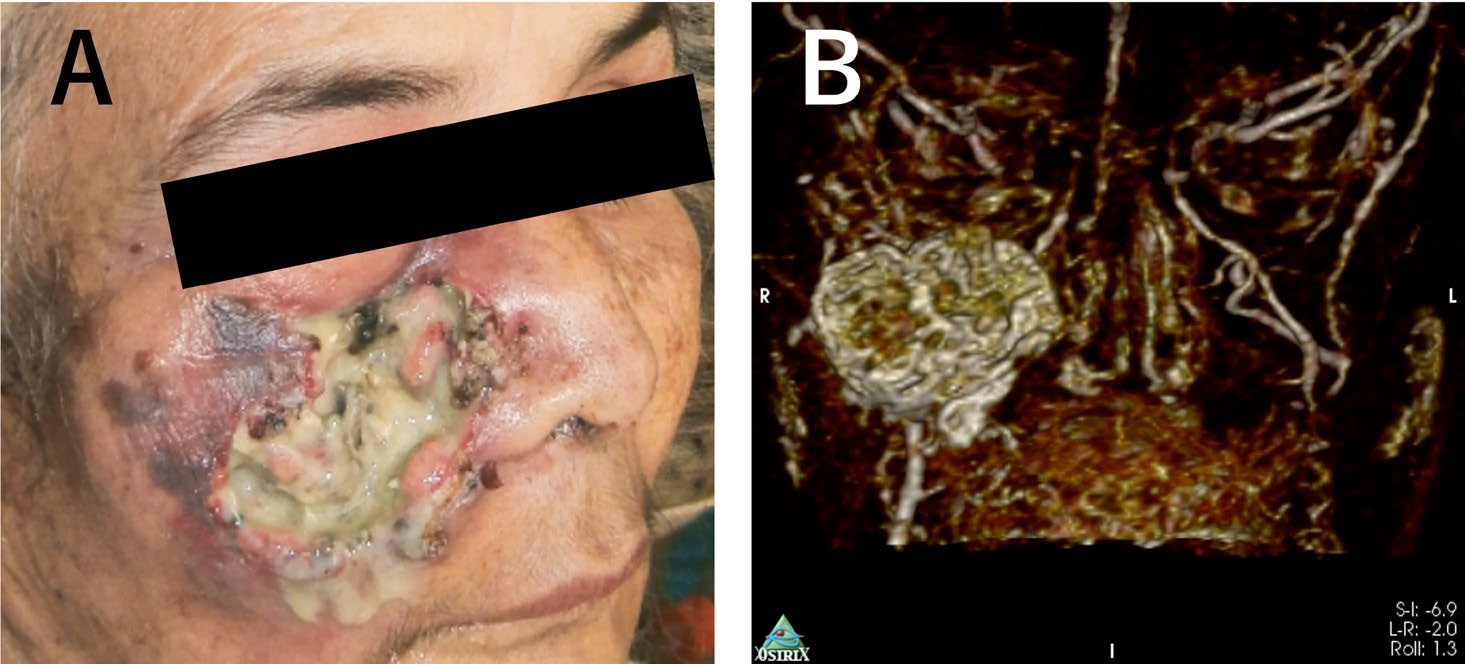
The Picture of wound area and MRI Image. (A) The infection had spread across the upper half of the patient's face during her first visit to the hospital. (B) Image of MRI analysis of the injured site indicated specific findings of wound myiasis.

A 64‐year‐old female patient from Bangladesh presented at our hospital in November 2021 with complaints of fever, bleeding from an infected site, ulceration, pus discharge, and severe pain. She reported a history of having a laceration on the cheek 1 month prior, hit by a sharpened wooden billet while watching Tip‐cat sport. The initial lesion was small (approximately 5 × 7 mm, as reported by the patient) but progressively enlarged. Upon presentation at the hospital, the pain and infection had spread across the upper half of her face (Figure 2A). On palpation, localized warmth, firm and tender swelling were observed. Examination of the wound revealed small grayish‐white maggots emerging from the wound. A complete blood count (CBC) showed leukocytosis with elevated neutrophil and eosinophil counts. Magnetic resonance imaging (MRI) analysis and reconstruction of the injured site revealed specific wound myiasis findings observed on the right side of the face and infraorbital region involving the skin and subcutaneous fat. The underlying muscle was inseparable from the lesion. The thyroid and parathyroid glands were not enlarged. There were no masses or calcifications, no evidence of displacement of the epiglottis or widening of the para‐ or retropharyngeal spaces, and no abnormalities or soft tissue masses in the naso‐oro‐hypopharynx (including the pyriform sinuses and posterior pharynx). The tracheal wall appeared smooth with no stenosis at the laryngotracheal junction. Cervical lymph nodes were also not enlarged (Figure 2B). Therefore, wound myiasis was diagnosed and it was caused by untreated trauma due to sports‐related injuries.

## 3 Investigations and Treatment

**FIGURE 3:**
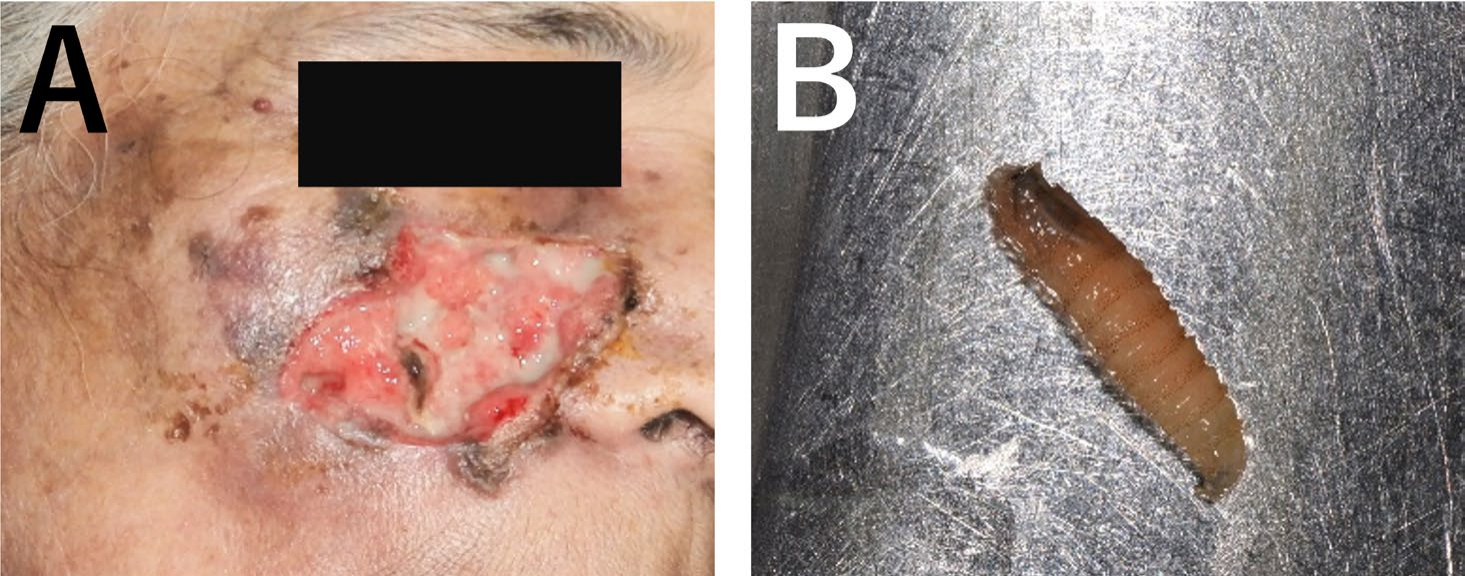
(A) After wound debridement and all maggots were removed. The wound was debrided daily under local anesthesia, then the maggots were completely removed using arterial forceps, followed by irrigation with saline and povidone‐iodine as antiseptic‐disinfectant agents for three consecutive days. (B) Maggots were 12–15 mm long, whitish, and without obvious body processes. The peritreme of the posterior spiracle is open, and the anterior spiracle has four to six lobes. These features are compatible with *Chrysomya bezziana* larvae.

First, turpentine oil, known to induce oxygen deprivation in wounds, prompting the maggots on the surface, was applied to the open wounds. Daily wound debridement under local anesthesia was performed, and the maggots were removed using arterial forceps, followed by irrigation with saline and povidone‐iodine (10% betadine solution) as antiseptic‐disinfectant agents for 3 consecutive days (Figure 3A). Approximately 70–80 extracted maggots were placed in a sealed container and sent to the laboratory for pathological analysis. The maggots were 12–15 mm long and whitish without obvious body processes. The peritreme of the posterior spiracle was opened, and the anterior spiracle had four to six lobes. These features were compatible with *Chrysomya bezziana* larvae (Figure 3B) [17]. The analysis showed that the maggots belonged to the *Chrysomya bezziana* species.

**FIGURE 4:**
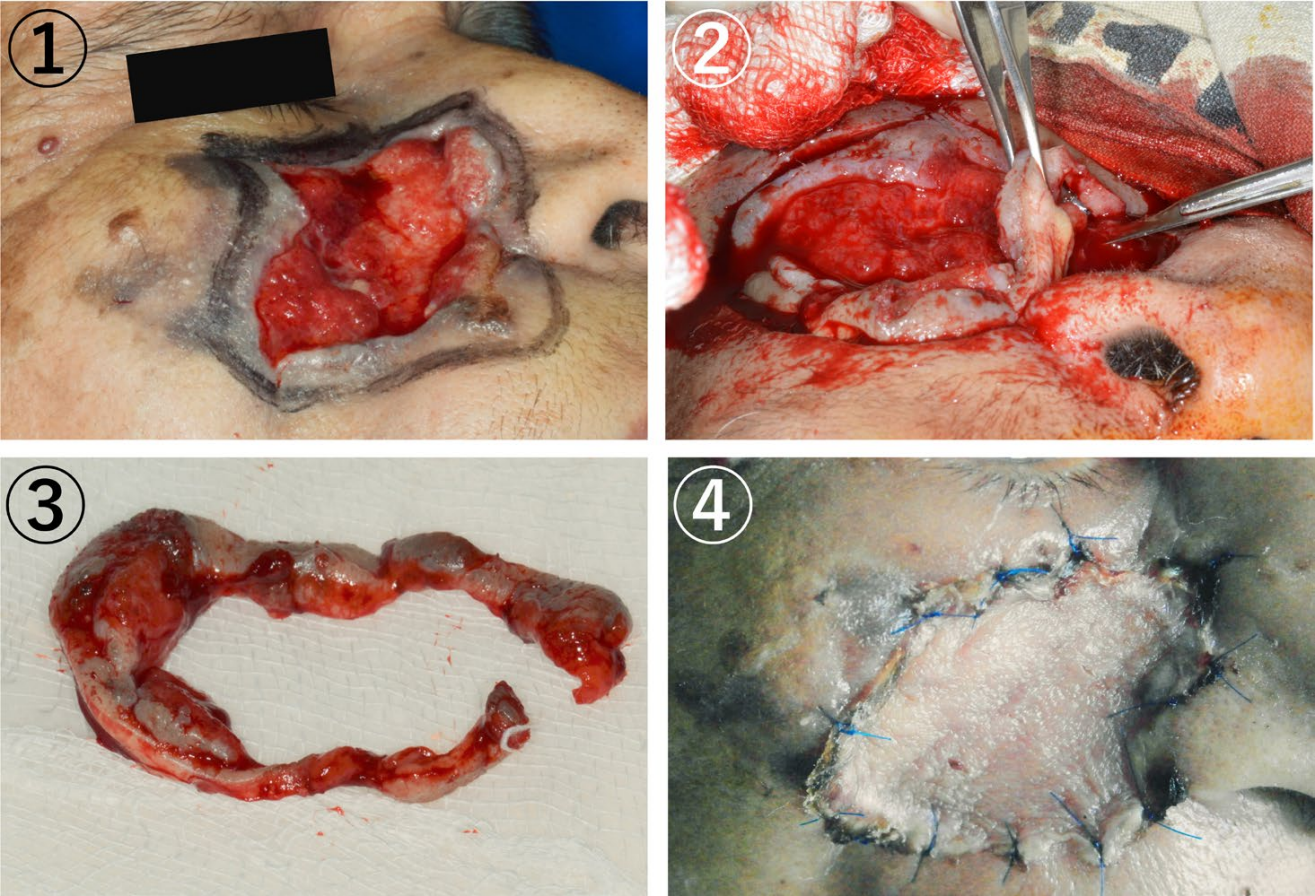
Debridement and skin graft. (A) An outline of the surgical area was made with an additional two millimeters of surrounding uninflamed tissue to ensure complete removal of the affected area. (B) During surgery, the affected tissue was excised and separate using a scalpel, scissors, and forceps. (C) The infected tissue was completely removed from the surgical site. (D) The skin graft was sutured over the surgical site.

**FIGURE 5:**
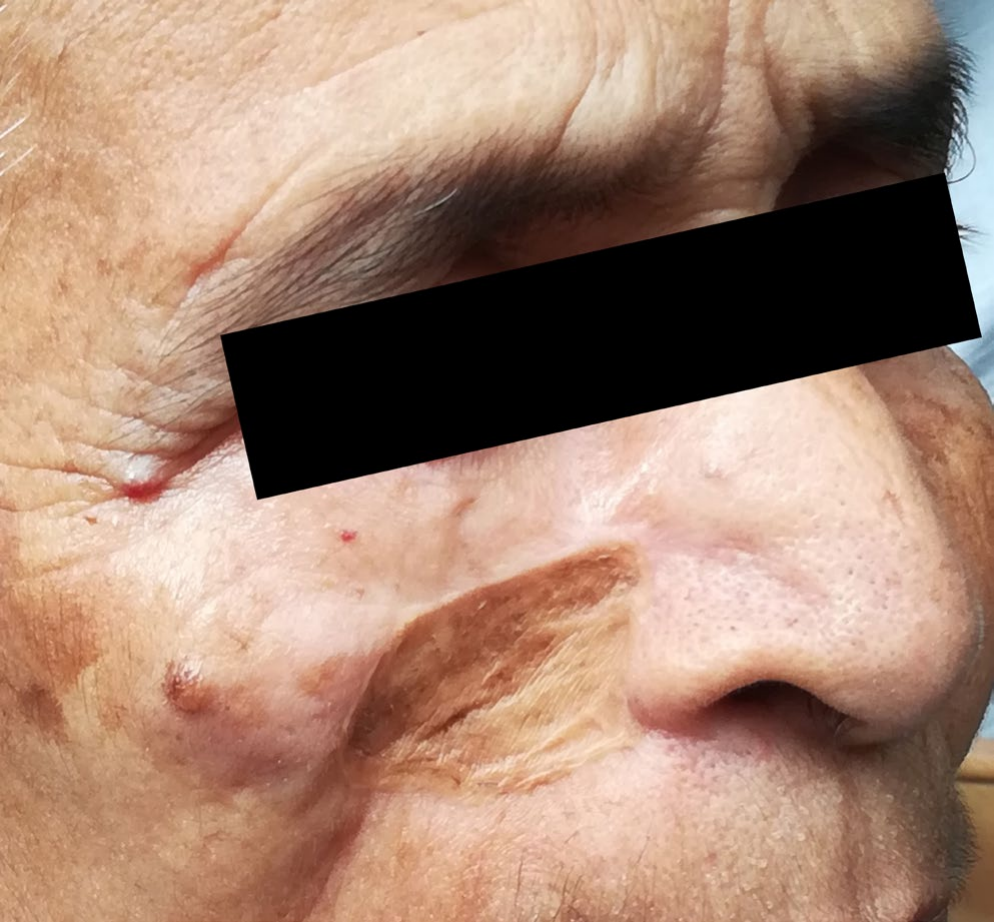
After healing the wound. The surgical wound healed 2 months post‐surgery.

The affected tissue was excised after completely removing the maggots, and a skin graft was performed using tissue from the left inner thigh (Figure 4). The patient was prescribed antibiotics and an analgesic for 5 days to prevent secondary infection and control the pain. The surgical wound healed in January 2022, 2 months post‐surgery (Figure 5).

The identity of the patient has been protected, and written informed consent was obtained from the patient.

## 4 Discussion

This case involved wound myiasis in a woman who watched Tip‐Cat‐Sport. Injury caused by a wooden tip struck by a player ultimately led to wound myiasis.

Spectators are prone to injuries in Tip‐Cat‐Sport because players may hit the wooden tip in the direction of the audience, as in baseball, where the spectators and players are in close proximity. The original wound size was small; however, inadequate infection prevention increased the likelihood of wound myiasis development. *Chrysomya bezziana* lays eggs in open wounds, which emit attractive odors that stimulate female insects to deposit eggs. The larvae invade the dermal layer and start feeding on the tissue using their oral hook, causing wound enlargement. This condition can prove fatal when the larvae invade body cavities or sinuses that cannot be directly visualized [18].

Low hygiene conditions and the lack of self‐care might have led to the onset of wound myiasis in this case. To prevent similar situations, it is crucial to increase awareness about the disease and establish preventive measures.

German entomologist Fritz Zumpt defined myiasis as “the infestation of live human and vertebrate animals with dipterous larvae, which at least for a period, feed on the host's dead or living tissue, liquid body substances, or ingested food [8]. Myiasis is clinically classified as primary or secondary. In the primary type, larvae feed on living tissue; in the secondary type, larvae feed on dead tissue [8]. Furthermore, myiasis is classified as accidental (larvae ingested along with food), semi‐specific (larvae laid on necrotic tissue in wounds), and obligatory (larvae affect the undamaged skin) myiasis, depending on the condition of the tissue involved. Based on the tissue involved, cutaneous myiasis is subdivided into creeping and furuncle. In the creeping type, larvae burrow through or under the skin, whereas in the furuncle type, larvae remain in one spot, causing a boil‐like lesion [8].

Flies causing myiasis belong to the order *Diptera*. The genera commonly reported are *Sarcophagidae*, *Calliphoridae*, *Oestridae*, and Muscidae from the *Diptera* order [16]. *Chrysomya bezziana*, the Old‐World screw‐worm fly, belongs to the genus *Calliphoridae*. It is an obligatory myiasis producer whose larvae develop only in living tissue, and human *Chrysoma bezziana* infestations are uncommon [16]. The species was first found in animal wounds in 2000, and the first human case was reported in 2003 [16]. *Chrysomya bezziana* is widely distributed across Asia, including Guangdong, Guangxi, Yunnan, and Taiwan. It is also found in tropical Africa, the Indian subcontinent, and Papua New Guinea [16].

Adult *Chrysomya bezziana* is a green or blue‐green fly widely distributed in tropical and subtropical countries. Under optimum conditions, *Chrysomya bezziana* development from eggs to adult flies can be completed in 18 days. The adult female fly lays eggs on live mammals and deposits approximately 150–200 eggs every 2 days on the wound in the body orifices. The eggs hatch after 12–18 h, and the first‐stage larvae, white in color, and 1.5 mm in length, emerge from the eggs and burrow into the wound or wet tissues. In approximately 2 days, the larvae molt into the second and third stages, 4–18 mm in length. After 5–7 days, the third‐stage larvae fall to the ground to pupate and transform into adult flies in approximately 7 days [19, 20].

Several prior reports document cases of wound myiasis. For example, a case study reported in 2009 involving *Chrysomya bezziana* included a 65‐year‐old woman with a cancerous wound [19]. This fly is native to tropical and subtropical areas [8], nevertheless, visitors including tourists, business professionals, and individuals participating in sports should take preventive measures if they sustain any injury in endemic areas, not only local residents.

Local application of several substances, including turpentine oil, larvicidal drugs such as Negasunt, mineral oil, ether, chloroform, ethyl chloride, creosote, saline, phenol, calomel, olive oil, and iodoform, can be used to remove larvae completely [21]. Turpentine is a toxic chemical that induces tissue necrosis. It can cause epithelial hyperplasia, hyperkeratosis, and ulceration when applied topically. However, the damage is reversible; the hyperplasia will only persist when the stimulus is applied continuously and regresses once it is withdrawn [21]. Turpentine oil can induce oxygen deprivation in the wound. This substance, called an asphyxiation drug, creates an anaerobic atmosphere within the wound, causing aerobic parasitic larvae to move to the surface, making their removal easier [8]. On emergence, they are easily removed manually with tweezers or forceps. A favorable prognosis is often achieved after thorough cleaning, debridement, and skin grafting.

Wound myiasis is uncommon worldwide. For example, the causative fly in this case, *Chrysomya bezziana*, mainly inhabits tropical and subtropical areas [8]. Consequently, awareness of this disease and treatment methods is often limited outside these regions. However, as international travel for business, tourism, and sports events increases, people who sustain injuries in these areas may develop wound myiasis and require treatment in their home countries. Moreover, as climate change progresses, the geographical range of wound myiasis may expand. Therefore, raising global awareness about wound myiasis is essential.

## 5 Conclusion

In this report, we describe a case of wound myiasis caused by a minor injury sustained by a tip‐cat sports spectator.

We hope this case report will contribute to increasing the knowledge about wound myiasis, including preventive measures and treatment methods, among those who previously had limited awareness of this condition.

## Author Contributions

**Jahan Esha Ishrat:** conceptualization, data curation, investigation, software, visualization, writing – original draft. **Kairi Hayashi:** conceptualization, data curation, visualization, writing – original draft. **Chowdhury Ruman Uddin:** conceptualization, investigation, methodology, software, visualization, writing – review and editing. **Chowdhury Nafees Uddin:** investigation, methodology, software, writing – review and editing. **Hiroshi Churei:** writing – review and editing. **Kenji Fueki:** project administration, writing – review and editing.

## Funding

The authors have nothing to report.

## Consent

Written informed consent was obtained from the patient to publish this report in accordance with the journal's patient consent policy.

## Conflicts of Interest

The authors declare no conflicts of interest.

## Data Availability

The data that support the findings of this study are available on request from the corresponding author. The data are not publicly available due to privacy or ethical restrictions.
